# Dysregulated coagulation system links to inflammation in diabetic kidney disease

**DOI:** 10.3389/fcdhc.2023.1270028

**Published:** 2023-12-08

**Authors:** Mengyun Xiao, Donge Tang, Shaodong Luan, Bo Hu, Wenyu Gong, Wolfgang Pommer, Yong Dai, Lianghong Yin

**Affiliations:** ^1^ Institute of Nephrology and Blood Purification, The First Affiliated Hospital of Jinan University, Guangzhou, Guangdong, China; ^2^ Shenzhen People’s Hospital/The Second Clinical School of Jinan University, Shenzhen, Guangdong, China; ^3^ Department of Nephrology, Shenzhen Longhua District Central Hospital, Shenzhen, Guangdong, China; ^4^ KfH Kuratoriumfuer Dialyse und Nierentransplantatione.V., Bildungszentrum, Neu-Isenburg, Germany; ^5^ The First Affiliated Hospital, School of Medicine, Anhui University of Science and Technology, Huainan, Anhui, China

**Keywords:** diabetic kidney disease, coagulation system, inflammation, protease-activated receptors, platelets, tissue factor, factor X

## Abstract

Diabetic kidney disease (DKD) is a significant contributor to end-stage renal disease worldwide. Despite extensive research, the exact mechanisms responsible for its development remain incompletely understood. Notably, patients with diabetes and impaired kidney function exhibit a hypercoagulable state characterized by elevated levels of coagulation molecules in their plasma. Recent studies propose that coagulation molecules such as thrombin, fibrinogen, and platelets are interconnected with the complement system, giving rise to an inflammatory response that potentially accelerates the progression of DKD. Remarkably, investigations have shown that inhibiting the coagulation system may protect the kidneys in various animal models and clinical trials, suggesting that these systems could serve as promising therapeutic targets for DKD. This review aims to shed light on the underlying connections between coagulation and complement systems and their involvement in the advancement of DKD.

## 1 Introduction

Diabetic kidney disease (DKD) represents the principal microvascular complication of both type 1 diabetes (T1DM) and type 2 diabetes (T2DM) (1) and stands as the primary cause of chronic kidney disease (CKD) and end-stage renal disease (ESRD) on a global scale (2). The clinical features of DKD in both T1DM and T2DM patients may be similar including increased albuminuria and a declined glomerular filtration rate. However, DKD progression varies in T1DM and T2DM (3). Although the prevalence of T2DM is higher than that of T1DM, a smaller proportion of patients progress to ESRD (3). Studies have suggested that T1DM patients are more likely to develop proteinuria 15–20 years after being diagnosed as having diabetes, with an incidence of 15%–40% (4). On the other hand, the incidence of proteinuria in T2DM patients varies from 5% to 20%, usually 15 years after the diagnosis (5). However, the situation in young T1DM and T2DM patients is quite different. Youngsters with T2DM present a higher prevalence of proteinuria and more rapid progression as well as a higher ESRD risk than those with T1DM (6). Despite the extensive research conducted, the exact mechanisms underlying DKD remain elusive.

## 2 The inflammation induced by the interactions between the coagulation system and the complement system in DKD

**Figure 1:**
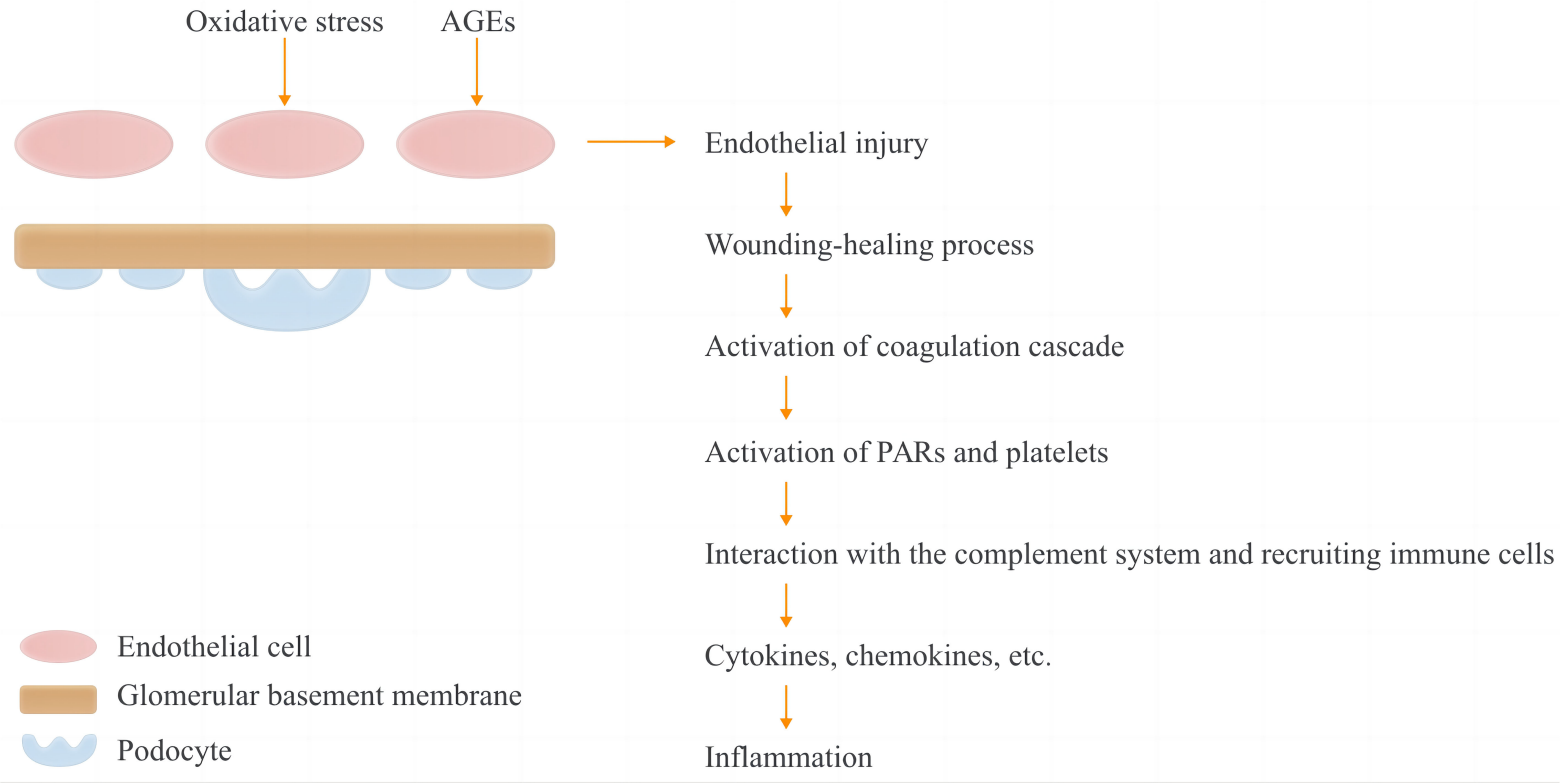
Hypothesis of activation of coagulation and complement systems and the subsequent inflammation in DKD patients. In T1DM and T2DM, increased oxidative stress and accumulation of advanced glycation-end products may cause endothelial injury. The coagulation and complement cascades are then activated as part of the initiation of the wound healing process as well as the first line of defensse against pathgen invasion. Protease-activated receptors (PARs) and platelets were activated by coagulation factors, and immune cells are recruited as the consequence of the interactions between coagulaiton and complement systems, with a subquent inflammation presenting increased cytokines and chemokines.

Glycation leads to the accumulation of advanced glycation end products (AGEs), causing intracellular oxidative stress and the deposition of extracellular hyaline, which results in the dysfunction of endothelial cells and kidney damage (7, 8). Consequently, the coagulation system is activated as the primary defense against invading pathogens and to initiate the healing process following renal injury (**Figure 1**) (9, 10).

Alterations in the coagulation system appear to be a common feature of T1DM and T2DM and may be associated with DKD progression (**Table 1**) (41, 42). For instance, increased levels of the von Willebrand factor, kallikrein, factor V (FV), factor VIIa (FVIIa), factor VIII (FVIII), factor X (FX), factor XI (FXI), prothrombin, and fibrinogen were observed in both diabetes types (15). Increased levels of the soluble tissue factor (TF), factor IX (FIX), factor XIIa (FXIIa), and factor XIII (FXIII) were only observed in T2DM (11, 17, 23). Furthermore, the elevated levels of TF, FXa, and thrombin possibly play critical roles in DKD (13, 43, 44). Alteration of coagulation factors between different diabetes types has been discussed in detail in another review (42). However, results from the existing clinical trials did not exclude varieties of interfering factors, such as age, race, and disease severity, and the effects of combined diseases.

**Table 1:** Expression of the coagulation factors, complement factors, and related regulators in plasma, urine, and kidney specimens of patients with diabetes or patients with DKD.

	Patients with diabetes			Patients with DKD		
	Plasma/serum concentration	Urinary concentration	Kidney	Plasma concentration	Urinary concentration	Kidney
Tissue factor	↑ (11)	↓ (12)	–	–	–	↑ (13, 14)
FX and FXa	–	↑ (15)	–	–	–	–
Thrombin	↑ (15, 16)	↑ (17, 18)	–	↑ (18)	↑ (17, 19)	–
Fibrin(ogen)	↑ (20, 21)	↑ (22)	–	↑ (23, 24)	–	↑ (25)
Platelets	↑ (26)	–	–	↑ (27)	–	–
C3	–	↑ (28)	–	↓ (29) ↑ (28)	↑ (28)	–
C5	↑ (28)	↑ (28)	–	↑ (28)	↑ (28)	–
C3a	–	–	–	↑ (30, 31)	↑ (30, 31)	–
C5a	–	–	–	↑ (31)	↑ (31)	↑ (32)
IL-1	↑ (33, 34)	–	–	↑ (35)	↑ (35)	–
IL-6	–	–	–	↑ (36, 37)	↑ (38)	↑ (39)
TNF-α	–	–	–	↑ (37, 40)	↑ (37, 40)	–

“-” refers to “not reported”

↑ refers to upregulated, ↓ refers to downregulated.

Addition to the coagulation factors, abundant evidence has indicated that the complement system, a critical component of the innate immune system, plays a crucial role in mediating inflammation during DKD progression (9, 28, 33, 35, 45). Alterations of complement factors, such as C3, C4, and C5 and their activated forms, are commonly observed (**Table 1**) and might be related to dysregulated coagulation factors in DKD (28, 33, 35, 36). While several reviews have individually summarized the roles of the coagulation and complement systems in DKD (8, 9, 26), few have explored the interconnections between these two systems and their collective contribution to DKD-related inflammation. This review aims to consolidate the current findings concerning the effects of interactions between the coagulation and complement systems on the advancement of DKD and explores potential therapeutic approaches targeting these systems.

**Figure 2:**
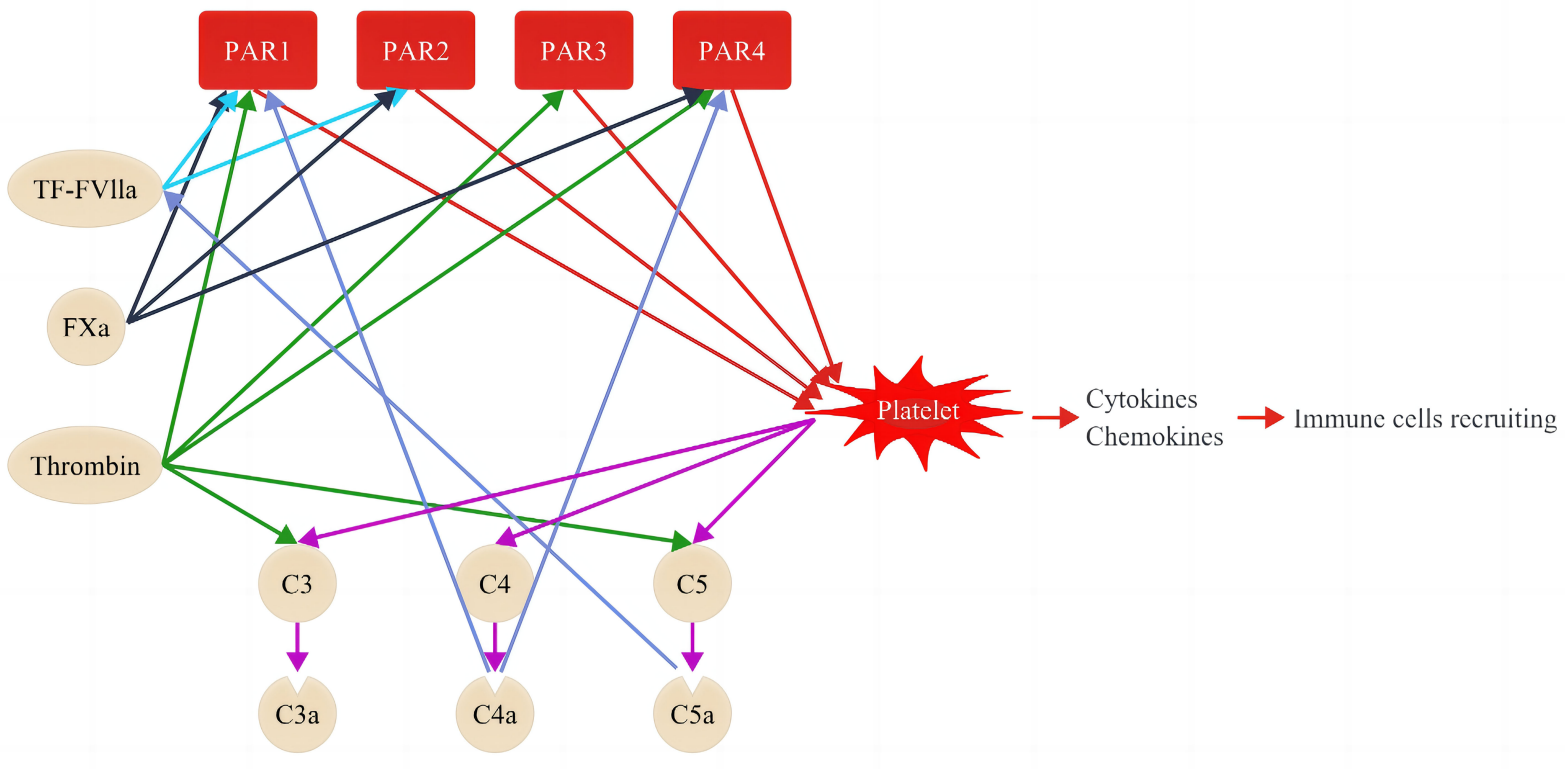
Coagulation cascade and its interactions with the complement system. Coagulation factors interact with complement systems through proteolytic activation of PARs, thereby causing sequential inflammation and upregulation of cytokines and chemokines. PARs comprise 4 members, namely PAR1, PAR1, PAR3, and PAR4. PAR activation by the coagulation factor is specific. For instance, the complex of TF–FVIIa activates PAR1 and PAR2 (light blue lines), Fxa activates PAR1, PAR2, and PAR4 (black lines), while thrombin activates PAR1, PAR3, and PAR4 (green lines). Coagulation factors can activate platelets through PARs (red lines) and induce sequential activation of complement factors, including C3, C4, and C5 (purple lines). The activated platelets promote inflammation by releasing cytokines and chemokines and recruiting sequential immune cells. The activated C4a and C5a, in turn, promote the activation of PAR1 and PAR4, and the complex of TF–VIIa, respectively (dark blue).

The elevated levels of coagulation molecules in diabetes are not only associated with a hypercoagulable state but also interact with the complement system, subsequently triggering inflammation in both diabetes and DKD (8, 46, 47). Studies have reported that the complement system is activated in patients with DKD and experimental DKD models (9, 45, 48, 49). The complement system can be activated through three pathways: the classical, lectin, and alternative pathways. Despite different initiations, these pathways converge into the same cascade by cleaving C3 and C5 through the corresponding convertases and sequentially activating subsequent steps to play a vital role in pathogen clearance (50). The role of complement activation in the progression of DKD has received considerable attention (9, 48). As clarified earlier and summarized in **Table 1**, the blood levels of coagulation were molecules elevated in patients with DKD (23, 51, 52). Some of these coagulation molecules such as thrombin, FXa, FXIa, and plasmin can directly convert C3 and C5 into their activated forms (C3a and C5a), thereby activating the complement cascades (50). Evidence of interactions between the coagulation and complement systems is reflected in the increased levels of C3(a) and C5(a) observed in both experimental models and patients with DKD (**Table 1**) (28, 30, 31). Additionally, complement factors influence the coagulation system (**Figure 2**), with C3 enhancing clot stability and increasing clot resistance to fibrinolysis by directly binding to fibrin (10). Moreover, aside from complement system activation, coagulation factors contribute to inflammation by activating cytokines such as interleukin (IL)-1, IL-6, and tumor necrosis factor (TNF)-α in DKD (9, 46, 47, 51, 53, 54).

The chronic inflammation induced by the activated coagulation and the complement systems, as well as cytokines, impair the filtration function of glomerulus (28). Kidney damage in DKD begins with glomerular hyperfiltration and culminates in renal fibrosis, encompassing glomerulosclerosis and tubulointerstitial fibrosis, which are consequences of the wound-healing response to renal injury.

## 3 Signaling pathways contribute to inflammatory fibrosis evolving the progression of DKD

Several signaling pathways, including Transforming Growth Factor-β1 (TGF-β1) (55), both canonical and noncanonical Wnt (56, 57), Notch (58), and the Nuclear Factor Erythroid-2-Related Factor 2-Kelch-Like ECH-Associated Protein 1 (Nrf2-Keap1) pathways (59), have been implicated in the fibrosis triggered by inflammatory responses in DKD (60).

TGF-β1 signaling, known to escalate collagen synthesis, impede extracellular matrix (ECM) degradation (61), recruit macrophages, and attract dendritic cells in DKD (62), is activated by pathogenic stimuli such as increased angiotensin-II, accumulating reactive oxygen species (ROS), and AGEs (55). Suppressing TGF-β1 signaling has shown to attenuate ECM accumulation and inhibit mesangial cell proliferation in both *in vitro* and *in vivo* studies (63, 64). Moreover, microRNAs like miRNA25 and miRNA29b are documented as renal-protective by curbing TGF-β signaling, thereby modulating inflammation and fibrosis in diabetic mice (65, 66). The canonical Wnt pathway is also a critical regulator of DKD progression. Over-activation of the Wnt/β-catenin signaling is linked to podocyte damage, which may be associated with inhibited podocyte de-differentiation (56, 67). However, inhibiting the Wnt/β-catenin pathway paradoxically led to severe proteinuria and amplified podocyte injury in diabetic mice (68, 69), suggesting both over-expression and deficiency of Wnt/β-catenin can damage podocytes. Moreover, noncanonical Wnt signaling, particularly through Wnt5a’s interaction with CD146, exacerbates renal inflammation in DKD, with blockade of this pathway ameliorating inflammation both *in vitro* and *in vivo* (57).

The Notch pathway, instrumental in cell proliferation, differentiation, and survival, is upregulated in DKD, influencing macrophage polarization (58). Notch signaling inhibitors in diabetic rodent models have demonstrated reduced Notch downstream gene expression, preserving podocyte markers and alleviating renal damage, such as glomerulosclerosis and podocyte loss (70).

Regarding the Nrf2-Keap1 pathway, Keap1 functions as an ROS sensor, prompting the liberation and activation of Nrf2, which then triggers transcription of cytoprotective genes (60). Bardoxolone, an inducer of the Nrf2-Keap1 pathway, has shown anti-inflammatory effects, improved eGFR, and decreased progression to renal endpoints in T2DM patients in the Phase 3 AYAME study (71).

Interactions among these signaling pathways also contribute to DKD progression. Notch signaling, for instance, modulates TGF-β and facilitates epithelial-to-mesenchymal transition (EMT) in human and cultured tubular epithelial cells (72). TGF-β1 may also potentiate Wnt/β-catenin signaling, promoting EMT in podocytes by upregulating Snail1, a transcription factor that advocates for EMT *in vivo* and *in vitro* (73). Moreover, ROS-induced Nrf2 signaling, essential for activating Notch signaling, participates in EMT as observed in cultured A549 cells (74).

Coagulation and complement cascades are hypothesized to serve as upstream events in DKD, instigating a plethora of changes that ultimately culminate in fibrosis. It is therefore essential to understand the changes of coagulation factors in DKD with respect to its critical roles involving the activation of the complement and the consequent inflammation-induced fibrosis in DKD. The correction of abnormality of the coagulation factors is beneficial for patients with DKD as it not only prevents thrombosis but also kidney failure (75, 76). The following sections will provide a summary of the dysregulations of critical factors in the coagulation system in DKD and their involvement in inflammation, along with their relevance to the progression of DKD.

## 4 Protease-activated receptors

PARs belong to the G-protein-coupled receptor superfamily and consist of four PAR proteins (PAR1–4) (40). PARs are expressed in several cells, including platelets, podocytes, mesangial cells, and distal tubules, and are involved in the progression of many renal diseases including DKD by activating inflammation via the induction of cytokines and chemokines (24, 41, 77–80). PARs can be proteolytic cleaved and activated by the specific coagulation and complement factors (40). For instance, the complex of TF and FVIIa (TF-FVIIa) activates PAR1 and PAR2; FXa activates PAR1, PAR2, and PAR4; thrombin activates PAR1, PAR3, and PAR4 (**Figure 2**) (41, 77, 81); complement factor C4a affects PAR1 and PAR4 activation (82). In turn, PARs can activate the innate immune system and inflammation by C3, C4, and C5 through the activation of platelets (9, 10, 30). Rahadian et al. observed increased expression of PAR1, PAR3, and PAR4, correlating with higher levels of inflammatory molecules such as monocyte chemoattractant protein (MCP)-1, intercellular adhesion molecule (ICAM)-1, and vascular cell adhesion molecule (VCAM)-1 in the aorta of streptozotocin (STZ)-induced diabetic mice (83). Notably, dabigatran, a thrombin inhibitor, was found to mitigate this upregulation of inflammatory molecules in these diabetic mice, which proved the bridging role of PARs in coagulation and inflammation (83). Additionally, PAR1 deficiency or administration of PAR1 inhibitors presented an anti-inflammation effect and anti-firosis by reducing the expression of MCP-1 and the TGF-β in renal inflammation in crescentic glomerulonephritis and obstructive kidney injury animal models (84, 85). Similarly, reduction of inflammatory genes were observed in injured kidneys with the administration of PAR2 inhibition (79, 86, 87).

Waasdrop et al. reported that PAR1 and PAR2 inhibitors improved kidney injury in a diabetic mouse model (88). The combined application of PAR1 and PAR2 inhibitors decreased albuminuria and glomerulosclerosis and downregulated the expression of inflammatory and fibrosis genes (88). Accordingly, inhibiting the regulators of PARs, including the coagulation factors TF and Factor Xa, can decrease inflammatory gene expression (89, 90). These findings indicated PARs and their regulators as potential therapeutic targets for inhibiting inflammation in DKD. However, data from clinical trials exhibiting renal benefits of PAR inhibitors in DKD patients are absent.

## 5 Platelets

Platelets are critical for maintaining the intact endothelium and coagulation (91), which contribute to fibrosis and inflammation (26, 83, 92, 93). Previous studies have shown that patients with DKD exhibit increased platelet distribution width (PDW), mean platelet volume (MPV), and increased platelet activity (26, 94) compared with those without DKD (95–97). Therefore, PDW and MPV are considered biomarkers of diabetic nephropathy (97). The mechanisms of hyperactivity of platelets in individuals with diabetes are complicated, including hyperglycemia, dyslipidemia, inflammation, and oxidative stress (98). Oxidative stress and inflammation increase the multiplication of platelets and increase the number of immature platelets (99). The receptors for the complement factors, C3aR and C5aR, are expressed on the platelet surface, indicating the potential activation of platelets via C3a and C5a (27). The dysfunctional endothelium in patients with diabetes and DKD leads to a reduction in nitric oxide and prostacyclin that regulate platelet aggregation (95, 99). The activation of platelets is via self-stimulation through the surface expression of CD36, protein kinase C, and coagulation factor II thrombin receptor-like 2, resulting in more platelet activation and the progression of inflammation and fibrosis in patients with DKD (96, 100).

The role of hyperactivity of platelets in the development of DKD has been determined via the activation of inflammation and fibrosis (26). Activated platelets induce inflammation by releasing various cytokines, such as TNF-α, P-selectin, TGF-β, fibronectin 1, ILs, and chemokines, such as platelet factor 4, CCL-2, chemokine (C-X-C motif) ligand 1, and chemokine (C-X3-C motif) receptor 1. These cytokines and chemokines are associated with the activation of neutrophils, macrophages/monocytes, and lymphocytes (101). Furthermore, platelets are also involved in inflammation via PARs (**Figure 2**). As mentioned above, TF and thrombin activate PARs on the surface of platelets (human platelets express PAR1 and PAR4, whereas mouse platelets express a PAR3/PAR4 complex (100) and cause substantial inflammation (**Figure 2**). Contrarily, platelets contain many types of cell growth factors, such as epidermal growth factor, TGF-β, and angiogenic factors, including PDGF, vascular endothelium growth factor, and fibroblast growth factor (26). These factors are involved in cell proliferation, mesangial expansion, stimulating profibrotic factor expression, and fibrosis in patients with DKD (102–104). As mentioned above, these factors target platelets owing to their multifactorial roles in the pathogenesis of DKD.

Recent studies involving experimental and clinical experiments investigated the role and renal effects of antiplatelet drugs in DKD (76, 91, 105–111) (**Table 2**). Different studies have reported the renoprotective effects of clopidogrel, cilostazol, and ticagrelor, which are platelet inhibitors, in a STZ-induced T1DM mouse model (76, 109–111). Specifically, clopidogrel ameliorated renal fibrosis by decreasing the levels of profibrosis-related proteins, including TGF-β, connective tissue growth factor, and fibronectin, in DKD mice (76). Cilostazol improved glomerular mesangial expansion, cell apoptosis, and ROS production in DKD mice (109, 110). Ticagrelor ameliorated kidney damage by reducing albuminuria, mesangial matrix expansion, and endothelial damage in DKD mice (111). However, the treatment effects of these platelet inhibitors were not investigated in T2DM models or clinical trials.

**Table 2:** Potential therapies in DKD targeting the coagulation or its regulators in both experimental stage and clinical trials.

Agent	Effect	Animal experiments	Clinical trials	Characteristics	Results	Limitations
E555	Inhibition of PAR1	eNOS^−/−^ * Ins2^C96Y/+^ mice (did not classify which type of diabetic model)	NA	Urinary albumin was 40–60 μg/mg creatinine; kidney pathology exhibited mesangial expansion.	Attenuation of glomerular injuries such as mesangial expansion and collagen IV deposition. Reduced renal expression of inflammatory and fibrotic genes (104).	The level of urinary albumin in this diabetic model was not as significant as that in other models.
FSLLRY	Inhibition of PAR2					
Clopidogrel	antiplatelet	STZ-inducd C57BL/6 mice (T1DM model)	NA	Urinary albumin was not reported in this study; kidney pathology exhibited an increased glomerular diameter in model mice.	Reduced expression of profibrosis-related proteins, such as TGF-β, connective tissue growth factor, and fibronectin (112).	Kidney damage was mild in this model and was not classical for mimicking DKD.
Cilostazol	antiplatelet	STZ-induced C57BL/6 mice with a high fat diet (T1DM model) (16) STZ-induced rats (T1DM model) (18)	NA	Urinary albumin was not reported in this study; kidney pathology exhibited an increased GBM thickness and mesangial expansion.	Reduced glomerular mesangial expansion, renal cell apoptosis, lipid accumulation, and ROS production (16, 18).	Kidney damage was mild in this model and was not classical for mimicking DKD.
Ticagrelor	antiplatelet	STZ-induced rats with unilateral nephrectomy (T1DM model) (113)	NA	Urinary albumin was 1000–1500 μg/24 h; kidney pathology exhibited mesangial matrix expansion, endothelial hypertrophy, and foot process fusion of podocytes.	Reduced albuminuria, mesangial matrix expansion, podocyte loss, and endothelial damage (113).	The role of non-platelet effects of ticagrelor, for instance, the inhibitory effects on endothelial cells and podocytes were not investigated and need to be explored in the future.
Sarpogrelate	antiplatelet	STZ-induced rats with spontaneously hypertension (T1DM model with hyper tension)	NA	Urinary albumin to creatinine ratio was 300–400 μg/mg creatinine; kidney pathology exhibited an increased glomerular diameter and mesangial expansion.	Sarpogrelate treatment inhibited the overexpressed proinflammatory and fibrotic signals such as increased collagen IV, α-SMA, and TGF-β in the kidney of diabetic animals (114).	Treatment effects of sapogrelate were not discussed in DKD mice without hypertension.
Sarpogrelate	antiplatelet	Db/db mice (T2DM model with hyper tension)	NA	Urinary albumin to creatinine ratio was 40–50 μg/24 h; kidney pathology displayed GBM thickening, mesangial expansion, and podocyte effacement.	Sarpogrelate significantly increased urinary albumin levels, improved glomerular damage, increased nephrin expression, and decreased VEGF expression in db/db mice (115).	Alterations of coagulation factors in DKD mice before and after treatment with sarpogrelate were not discussed.
Sarpogrelate	antiplatelet	NA	Retrospective clinical study (14440 patients with T2DM)	Urinary albumin to creatinine ratio was 92–140 mg/24 h; kidney pathology exhibited GBM thickening, mesangial expansion, and podocyte effacement.	Incidence and progression of DKD reduced. However, the estimated glomerular filtration rate and urinary albumin to creatinine ratio were not significantly changed after treatment (116).	Incomplete data acquisition. Data from a prospective study would be required in the future.
Beraprost sodium	antiplatelet	NA	Prospective clinical study (patients with T2DM)	Overall, 102 patients aged 40–60 years with type II DKD; urinary albumin/creatinine was ≥30 mg/mmol or ≤300 mg/mmol; results of kidney pathology were not shown.	Reduced levels of fibrin, D-dimer, platelets, cystatin C, β2-microglobulin, and α1-microglobulin, and the urinary albumin/creatinine ratio (117).	Data from multicenter clinical randomized controlled studies would be required in the future.
Beraprost sodium	antiplatelet	NA	Multicenter prospective, randomized, double-blind, placebo-controlled clinical trial (patients with T2DM)	Fifty-two patients aged 19–70 years with type II DKD; urinary albumin to creatinine ratio was 30–299 mg/24 h or albumin in spot urine was 30–299 μg/mg creatinine. However, the results of kidney pathology were not shown.	Systolic blood pressure was significantly decreased by BPS. However, albuminuria was not significantly different after BPS treatment (118).	Variables such as waist circumference and body mass index, and medications that affect primary outcomes were not well-controlled in this study.
Anti-TF antibody	Inhibition of TF	STZ induced eNOS^-/-^ * mice (type I diabetic model) with a high fat diet)	NA	Urinary albumin: 100–200 μg/day; kidney pathology showed severe glomerulosclerosis, tubulointerstitial fibrosis, and GBM thickening.	Reduction in the renal mRNA levels of inflammatory and fibrotic genes (76).	The role of anti-TF antibody in the reduction of albuminuria and improvement in renal injury were not mentioned.
Fondaparinux	Inhibition of FXa	db/db mice (T2DM model)	NA	Urinary albumin was 1650 ± 750 mg/mg creatinine in db/db mice at week 26; kidney pathology displayed glomerular hypertrophy and fibrin deposition.	Reduction in albuminuria, urinary excretion of inflammatory and fibrotic proteins, improvement in glomerular hypertrophy, fibrin deposition (119).	FXa activity in the kidney after treatment with the FXa inhibitor was not reported.
Edoxaban	Inhibition of FXa	eNOS^−/−^ * Ins2^C96Y/+^ mice (did not classify which type of diabetic model)	NA	Urinary albumin was 186 ± 20 μg/day; kidney pathology exhibited mesangial expansion, glomerulosclerosis, and GBM thickening.	Amelioration of mesangial matrix proliferation. Reduced renal expression of inflammatory genes (78).	FXa activity in the kidney after treatment with the FXa inhibitor was not reported.

PAR refers to protease-activated receptor; T1DM refers to type 1 diabetic model; T2DM refers to type 2 diabetic model; NOS refers to NO synthase; STZ refers to streptozotocin; TGF-β refers to transforming growth factor beta; GBM refers to glomerular basement membrane; ROS refers to reactive oxygen species; α-SMA refers to alpha smooth muscle actin; VEGF refers to vascular endothelial growth factor; TF refers to tissue factor; NA refers to not applicable.

Sarpogrelate, a selective competitor of 5 hydroxy-tryptamine-2A that causes antiplatelet aggregation, exerted renoprotective effects in both type I and type 2 DKD mice by inhibiting the overexpression of collagen IV, α-SMA, and TGF-β, which are involved in inflammation and fibrosis (107, 108). Furthermore, a retrospective study of 14440 T2DM patients receiving sarpogrelate treatment exhibited decreased incidence and progression of DKD (106). These results demonstrated that sapogrelate caused a significant renal improvement in DKD, especially in those with mild proteinuria (**Table 2**) (106–108). However, data from large prospective clinical trials evaluating the protective effects of sarpogrelate against DKD will be required.

Beraprost sodium (BPS) is a drug that exerts an antiplatelet aggregation effect. Two prospective clinical studies have reported BPS to be effective in reducing inflammation and fibrosis in patients with type 2 DKD (91, 105). In one prospective clinical study with 102 DKD patients, the efficacy of BPS combined with alprostadil was investigated, which are two antiplatelet aggregation drugs (91). The evidence of renoprotective effects of BPS due to anticoagulation was presented as a significant decrease in the urinary albumin/creatinine ratio, cystatin C, β2-microglobulin, and α1-microglobulin, with a simultaneous reduction in FIB, D-dimer, and platelet levels (91). More importantly, the TNF-α level also decreased after treatment with BPS, thereby indicating a decrease in inflammation in DKD patients after antiplatelet treatment (91). In another prospective study, systolic blood pressure decreased in 52 patients with type 2 DKD; however, albuminuria exhibited no significant difference after a 24-week administration of BPS compared with placebo treatment (105). The small sample size and the different patient age-related inclusion criteria may be the reasons for the inconsistent results of these two studies. Therefore, large prospective clinical trials are warranted to further confirm the protective effects of BPS in patients.

## 6 Factors of coagulation cascades

### 6.1 The relationship between TF levels, NOs, and inflammation markers

TF, also known as factor III, is an upstream molecule of the extrinsic coagulation system. The downstream cascade of extrinsic coagulation is activated when FVIIa binds to TF (37). TFs are expressed by podocytes and parietal epithelial cells in human kidneys (33, 34). The circulating TF levels were elevated in patients with CKD compared with healthy controls and were negatively correlated with a lower estimated glomerular filtration rate (eGFR) (35). As the most prevalent reason for CKD, DKD is associated with TF overexpression and subsequently leads to hypercoagulability (41). An *in vitro* study using human renal mesangial cells reported upregulated expression of TF by AGE and glucose in a concentration-dependent manner (36). Furthermore, the upregulation of renal TF expression was confirmed in T1DM mice in which diabetes was induced by STZ) (40).

The complement factor C5a can aggravate the overexpression and overactivity of TF (120, 121) and further activate platelets, which can promote thromboinflammation sequentially by affecting TNF-α and IL-6 production and secretion (122). In turn, the upregulated TNF-α promotes TF expression (123). Furthermore, TF can induce inflammatory cytokines by mediating PAR 1 activation (100) (**Figure 2**). Recently, Yuji Oe et al. conducted a series of studies to elucidate the roles of TF in an experimental DKD model that lacked eNOS and had an Akita mutation in the Ins2 gene (eNOS^−/−^ * Ins2^C96Y/+^ mice) (89, 124). They reported that renal TF overexpression and overactivity were positively correlated with the increased renal expression of inflammatory genes such as IL-6, ICAM-1, TNF-α, and MCP-1, as well as the increased plasma concentration of IL-6 (124). On the other hand, upregulated renal TF expression was also positively correlated with the increased concentration of urinary albumin excretion, glomerular fibrin deposition, and glomerulosclerosis (41). Elevated TF expression synergistically increased when the *eNOS^−/−^* mice were administered a high-fat diet (13, 41, 125). The administration of anti-TF neutralizing antibodies, which inhibited TF activity up to 70%, decreased the expression of inflammatory and fibrosis marker genes, including TNF-α, monocyte chemoattractant protein-1 (CCL2), and TGF-β, in the kidneys of eNOS-deficient DKD mice carrying the Akita mutation in the insulin 2 gene (*eNOS^-/-^* mice) (13). These results confirmed that TF elevation caused inflammation in the kidneys, thereby leading to DKD progression, and thus, TF can be used as a potential therapeutic target (13). However, these findings were based on experimental DKD, and data from clinical studies would be required in the future. The mechanisms underlying TF-activated inflammation and fibrosis in DKD should be investigated more specifically.

FX and its active form FXa are essential for hemostasis progression, including both the extrinsic and intrinsic pathways (117). Several studies have used different animal CKD models and revealed elevated FXa levels (116, 118). FXa increases the levels of inflammatory molecules such as IL-1 and TNF-α (116) through the PAR-dependent pathway (44). The FXa inhibitor edoxaban exhibited renoprotective effects by attenuating macrophage infiltration and inflammatory molecules in a unilateral ureteral obstruction model (116). As a regulator of PARs, FXa primarily activates PAR1 and PAR2 (112, 116).

In a previous study, the roles of FXa and PARs were investigated in *eNOS^-/-^* mice (44). By using the same model, researchers have reported increased infiltration of TF-expressing macrophages in glomeruli. TF activated FX, which was supported by the evidence of increase in FX mRNA levels in the renal tissue, urinary FXa activity, and FX-expressing macrophages (9). The overactivated FXa caused the subsequent activation of PARs and inflammation in DKD patients. The renoprotective effects conferred through edoxaban-induced inhibition of FXa activity in DKD mice were related to amelioration of the expression of PARs and proinflammatory genes (9, 44), and caused a decrease in urinary albumin excretion, plasma cystatin C levels, and mesangial proliferation (9, 44). In the same study, researchers observed that FXa inhibition presented similar anti-inflammatory effects in mice with PAR2 deficiency. Taken together, these results suggest that FXa-induced inflammation in DKD mice occurs in a PAR2-dependent manner (44). Moreover, stimulation of endothelial cells and podocytes with FXa and PAR2 agonists, respectively, increased the production of proinflammatory factors of interleukin-8, MCP1, and PAI1 *in vitro* (115). However, these studies did not elucidate the inhibition efficiency of FXa inhibitors against FXa activity and expression in kidney tissues.

Although the evidence of the renoprotective effects of the FXa inhibitor in DKD is still absent, a meta-analysis offered some hints of the safety and efficacy of orally administered edoxaban in patients with both atrial fibrillation (AF) and CKD (114). Compared with warfarin, edoxaban presented an inferior effect in terms of anticoagulation, and a significantly lower risk of bleeding, ischemic stroke, and mortality in patients with AF (114). Furthermore, results revealed that edoxaban led to better improvements in kidney function than warfarin and a significant reduction in the bleeding risk in patients with both AF and CKD (114). Large prospective clinical trials investigating the safety, efficacy, and renoprotective effects of FXa inhibitors in DKD patients are warranted.

### 6.2 Thrombin

Thrombin is a central hemostasis regulator owing to its function in fibrin formation and platelet activation (**Figure 2**). Furthermore, thrombin is probably involved in the complement system by activating both C3 and C5 and can induce inflammation via PARs (**Figure 2**). Serum thrombin level, an indicator of cardiovascular morbidity and mortality, is increased in patients with T2DM and correlates with albuminuria (15, 16). This suggests that thrombin plays an important role in the development of kidney failure (18). In many patients with diabetes, particularly in those with T2DM, obesity can not only promote hypercoagulation but also induce chronic inflammation (51). Past studies have confirmed that the thrombin inhibitor dabigatran etexilate ameliorates obesity development and inflammation severity in a high-fat animal model (51, 113).

In previous studies, plasma and urinary thrombin levels were measured in patients with DKD (17, 18). One cross-sectional study involving 160 patients with T2DM revealed that the area under the curve of thrombin significantly differed between patients with T2DM with or without albuminuria (18). Furthermore, abnormalities in thrombin generation were probably the reason for macrovascular disease in patients with DKD (18). In another study, urinary thrombin levels were measured in 118 patients with type 2 DKD; a significant association was observed between decreased eGFR and increased proteinuria (17).

Thrombin triggers inflammatory factors such as TNF-α, IL-1, and IL-6 by cleaving PAR1 (126). In turn, the PAR1-dependent signaling pathway activates endothelial cells, leading to the exposure of P-selection and expression of PAI-1 and other inflammation cytokines (126). These PAR-1-induced endothelial effects can further promote thrombosis by regulating platelet adhesion (127). Considering the prominent role of thrombin in coagulation and inflammation, it can serve as a suitable therapeutic target for DKD by inhibiting the hypercoagulable and hyperinflammatory states. Dabigatran, a direct thrombin inhibitor, was approved by the European Medicines Agency in 2008 and then by the Food and Drug Administration in 2010 for the treatment of venous thromboembolism (VTE) as well as for use in the prevention of VTE after hip and knee arthroplasty (128). However, due to the potential risk of renal injury caused by dabigatran, this drug has not been recommended for patients with CKD stage 5 or patients undergoing dialysis (129). Thus, the anti-inflammatory effects of dabigatran remain unelucidated in animal models or clinical trials of DKD.

### 6.3 Fibrinogen and fibrin

FIB, the most abundant molecule of the plasma coagulation system, is the main protein involved in the formation of blood clots (130). In addition to its function in coagulation, FIB is also an inflammatory indicator because its levels rapidly increase in the acute phase of inflammation (131). In an animal model of FIB deficiency, macrophage adhesion, and cytokine production decreased during inflammation, indicating an interaction between coagulation and inflammation (132). Overexpressed FIB in the circulation may result in FIB deposition in the kidneys and the promotion of tubulointerstitial or glomerular fibrosis (78, 133), by stimulating renal cells to secrete chemokines and cytokines (79).

In a previous study, urinary FIB levels were measured in patients with CKD with different etiologies; 101 patients with biopsy-based DKD were included (22). Results revealed that urinary FIB is correlated with the risk of progression of CKD to ESRD (22). Two additional retrospective cohort studies have reported elevated plasma/serum FIB levels in patients with DKD than in those with T2DM but without DKD (24, 52). Fibrin(ogen) protein glycation enhances the crosslinking of fibrin and FXIIIa. Furthermore, it increases the resistance of patients with diabetes to fibrinolysis (77, 84), possibly leading to an increase in FIB levels. Elevated serum FIB level is associated with a higher risk of progression to ESRD (24). Another study with a more significant number of patients, i.e., 1022 patients with DKD and 1203 patients with T2DM, revealed that elevated serum FIB levels and the ratio of FIB to albumin in patients with DKD are independent risk factors for DKD progression in patients with T2DM (23). However, most of the current studies that have suggested elevated plasma/serum FIB levels as a predictor of DKD are retrospective. Therefore, additional evidence from prospective studies is warranted. Furthermore, the mechanism by which elevated FIB participates in DKD progression and whether it is related to inflammation-induced fibrosis should be investigated.

## 7 Conclusion

The activation and elevation of coagulation factors as well as complement factors were widely observed in type 1 and type 2 DKD models and patients (**Table 1**). Results from current studies have suggested that the interactions between coagulation and the complement system can lead to inflammation and play a critical role in the pathogenesis of DKD, such as inducing glomerular and renal interstitial fibrosis (51). Therefore, targeting coagulation factors, such as TF, FXa, thrombin, fibrin, and platelets that induce inflammation can be potential therapeutic approaches for preventing the progression of DKD. The renoprotective effects and decreased inflammation have been reported in studies using anti-TF neutralizing antibodies (76), FXa inhibitors (44), inhibitors of PAR1 and PAR2 (134), and antiplatelets in type 1 and type 2 DKD models and patients. Although thrombin plays key roles in coagulation cascades and activation of the complement system as well as inflammation, thrombin inhibitors may not be suitable for DKD patients due to their potential risk of renal injury (129, 135).

As summarized in **Table 2**, the renoprotective effects of coagulation factor inhibitors were investigated in type 1 or type 2 DKD. Among these coagulation inhibitors, the renal effects of antiplatelet drugs have been extensively studied. Of them, sarpogrelate was studied in a retrospective clinical study (108), and prospective studies have reported promising treatment effects of BPS in patients with type 2 DKD (76, 107). However, the treatment effects of these antiplatelet drugs in patients with type 1 DKD need to be investigated in the future. The conclusions of anticoagulants were essential for reducing inflammation and fibrosis in DKD were drawn on the basis of the results of a few studies, and the currently included studies had some limitations (13, 43, 44, 75, 76, 107–111, 119, 134, 136). First, these included studies had investigated the roles of coagulation–complement factors in patients with type 1 or type 2 DKD, or solely with experimental models. Few studies have evaluated the roles of coagulation–complement factors in a combination of both types of DKD, or the combination of both *in vivo* studies and clinical trials. Second, the currently included clinical trials had investigated the treatment effects of anticoagulation using antiplatelet drugs. Beyond these clinical trials, only two prospective studies have investigated the reno-protective effects of anticoagulation with a small sample size (76, 107). Therefore, large prospective clinical studies evaluating the effects of anticoagulation in patients with type 1 or type 2 DKD are warranted. Based on the studies included in this review, we noted that dysregulated coagulation is common in patients with type 1 DKD and type 2 DKD. Patients with type 1 or type 2 DKD basically receive common anticoagulation treatments with glycemia control, weight control, plasma lipid level-reducing, and finally, antiplatelet drugs (42). However, the evidence or studies discussing the differences in the renal effects of anticoagulation drugs between type 1 and type 2 DKD are currently absent and will need to be investigated in the future. The additional animal experiments and clinical trials are required to reach the final goal of individual care for each patient with DKD, which can achieve the best antithrombotic and anti-inflammatory effects, and safety. Several drugs targeting the thromboinflammation in DKD patients could be interestingly attempted in clinical trials. For instance, small molecules such as E555 and FSLLRY, which are antagonists of PAR1 and PAR2, respectively, exerted renoprotective effects in animal models (109, 134). However, the efficacy and safety of these drugs need to be further investigated in the future. Furthermore, investigating the effects of the FXa inhibitor edoxaban in reducing inflammation and improving kidney damage in DKD patients will be interesting because of its renal benefits in CKD patients (114).

## Author contributions

MX: Conceptualization, Visualization, Writing – original draft. DT: Writing – review & editing. SL: Writing – review & editing. BH: Funding acquisition, Writing – review & editing. WG: Funding acquisition, Writing – review & editing. WP: Writing – review & editing. YD: Funding acquisition, Supervision, Writing – review & editing. LY: Funding acquisition, Supervision, Writing – review & editing.
